# Spatial and Temporal Movements of Free-Roaming Cats and Wildlife in Two Local Government Areas in Greater Sydney, Australia

**DOI:** 10.3390/ani13101711

**Published:** 2023-05-22

**Authors:** Isabella J. L. Davey, Mark E. Westman, Dominique Van der Saag, Gemma C. Ma, Brooke P. A. Kennedy

**Affiliations:** 1 School of Life and Environmental Sciences, Faculty of Science, The University of Sydney, Camperdown, NSW 2006, Australia; idav3138@uni.sydney.edu.au; 2 School of Veterinary Science, Faculty of Science, The University of Sydney, Camperdown, NSW 2006, Australia; mark.westman@sydney.edu.au (M.E.W.); dominique.van.der.saag@sydney.edu.au (D.V.d.S.); gemma.ma@sydney.edu.au (G.C.M.); 3 Royal Society for the Prevention of Cruelty to Animals (RSPCA) NSW, Yagoona, NSW 2199, Australia; 4 School of Environmental and Rural Science, University of New England, Armidale, NSW 2351, Australia

**Keywords:** cat, behaviour, *Felis catus*, free-roaming, monitoring, One Health, outdoor, veterinary science, wildlife

## Abstract

**Simple Summary:**

Free-roaming cats (domestic and feral) are known to negatively impact wildlife numbers in Australia and can also affect human health via zoonotic diseases. To help learn more about their outdoor movement, free-roaming cats in two local government areas (LGA) in New South Wales (NSW), Australia, Campbelltown (CT) and the Blue Mountains (BM), were monitored in this study using two different data gathering methods. The BM LGA contains high levels of National Park land compared to CT LGA, however, Campbelltown’s human population is more than double and its population density is approximately ten times higher than the BM region. Motion-capture cameras were installed on 100 properties (50 per LGA) to capture all animal movements over a two-month period and transect drives along pre-determined routes through both areas (four per LGA) were completed to directly observe free-roaming cats in residential areas. The results showed higher numbers of free-roaming cats in CT LGA, and higher levels of wildlife in the BM LGA. Free-roaming cats were seen roaming throughout the day. This data provide a baseline for the Royal Society for the Prevention of Cruelty to Animals (RSPCA) NSW Keeping Cats Safe at Home program which is designed to reduce free-roaming cat numbers.

**Abstract:**

Free-roaming cats pose a risk to their own health and welfare, as well as to the health and welfare of wildlife and humans. This study aimed to monitor and quantify area-specific free-roaming cat movement. Two local government areas (LGAs) in Greater Sydney were included, Campbelltown (CT) and the Blue Mountains (BM). Motion-capture cameras were installed on 100 volunteer properties (50 per LGA) to indirectly capture animal movements over two months. Transect drives were completed eight times (four per LGA) to directly observe roaming cats in residential areas. The cameras and transects both identified higher free-roaming cat numbers in CT (density of 0.31 cats per ha, resulting in an estimated abundance of 361 cats in the 1604 ha of residential area) than the BM (density of 0.21 cats per ha, resulting in an estimated abundance of 3365 cats in the 10,000 ha of residential area). More wildlife events were captured in the BM (total = 5580) than CT (total = 2697). However, there was no significant difference between CT and the BM for cat events (*p* = 0.11) or wildlife events (*p* = 0.32) observed via the cameras. Temporally, cats were observed via the cameras throughout the entire day with peaks at 9:30 am and 8:00 pm in the BM, and 7:00 am and 12:00 pm in CT. Overlaps in activity times were recorded for free-roaming cats with bandicoots (BM), possums (BM), and small mammals (BM and CT). This study demonstrates that camera monitoring on private property and transect drives are useful methods to quantify free-roaming cat abundance to inform cat management interventions.

## 1. Introduction

The domestic cat (*Felis catus*) is the largest species of companion animals globally, with more than half a billion cats estimated worldwide [1]. Whilst labelled a domestic species, *Felis catus* has also proven to be capable of surviving in the wild and is considered an introduced species in many countries including Australia [2]. According to the Royal Society for the Prevention of Cruelty to Animals (RSPCA) in Australia, cats that are ‘unowned, unsocialised, have no relationship with or dependence on humans and reproduce in the wild’ should be defined as ‘feral’ cats [3]. Globally, *Felis catus* are non-randomly distributed across time and space, and their presence varies from 0.1 to >1000 cats per square kilometre [4]. Higher density cat populations are typically seen in and around urban environments [4]. In Australia, there are approximately 4.09 million owned cats in 30% of households, and approximately 2.8 million feral cats, covering more than 99.8% of Australia’s surface area [5,6,7,8].

Domestic cats (owned, semi-owned, and stray), and feral cats, may be known as ‘free-roaming’ (not confined to a person’s yard or house), whilst some owned cats are considered ‘non-roaming’ as they are contained and/or strictly indoors-only [9,10]. In a review of six Australian studies of owned cats, it was estimated that 71.1% were able to roam outside and could be classified as free-roaming, with the remaining considered as non-roaming [11]. Cats are enticed to roam outdoors by interesting scents, discovery, climbing, and hunting [12]. A cat’s roaming range is the area it most routinely travels and this varies immensely; it is affected by the environment (urban or rural) and the cat’s ownership status (owned or unowned) [13]. Each cat’s range contains different sections, with the outermost area used for food patrol, and the inner areas for defending territory and as personal space to play, eat, and sleep [12]. Traditionally in Australia, cat ownership has permitted free-roaming, with owned cats allowed to roam at night having larger ranges and roaming further than those only permitted to roam during the day [2,14].

Outdoor roaming can present risks to cats including injury from vehicles and other animals, poisoning, and cruelty from humans [4,12]. Cats that live outdoors can also experience starvation and high kitten mortality [15]. Not only can pathogens, such as feline immunodeficiency virus (FIV) and feline leukemia virus (FeLV), be passed intraspecies [15,16], but diseases can also be spread interspecies including with mammals (both wild and domestic), birds, and humans [13,17,18,19]. Cats also compete with native predators, alter prey behaviour, and burden wildlife rehabilitation and native species’ reintroduction [17,20].

All felids are obligate carnivores and most are generalist ambush predators [6]. Although domestic cats are known to eat all vertebrate classes and arthropods [17], they primarily hunt small mammals [21]. Domestic cats are also facultative specialists, meaning that they switch between prey when more profitable prey becomes available, particularly with seasonality [21]. Like their ancestors, domestic cats are crepuscular, hunting at dawn and dusk, and during the night [13]. Whilst owned cats rely on their owners for food, all free-roaming cats (including owned cats) can display normal predatory and hunting behaviours. Consequently, contained cats need enrichment for play behaviours to help stimulate and satisfy this innate predatory behaviour [6,12]. Free-roaming cats are the main threat to almost 8% of critically endangered mammals, birds, and reptiles globally, and are considered to have contributed to at least 14% of global extinctions [9,22]. In Australia, mammals make up most of their prey, followed by reptiles and birds in areas with fewer mammals [23]. Although there is no consensus on the exact numbers, some researchers have estimated that owned and unowned (feral and stray) free-roaming domestic cats kill 61 million birds and 53 million reptiles in Australia annually [23,24]. Despite the possibility that cat numbers and therefore predation may be over-estimated [25], the threat of cat predation on wildlife has led to them being listed amongst 100 of the world’s worst invasive species [9]. The extent and significance of free-roaming cats and their impact on wildlife destruction are an ongoing debate [4]. With the rise of conservation biology as a discipline, the role and impact of free-roaming cats on endangered and threatened species has garnered more attention [26]. Of particular concern are islands and areas with naive and susceptible prey species that have evolved in the absence of mammalian predators [9,17].

Research to date reporting the impact of free-roaming cats, both in Australia and overseas, is limited and has mainly focused on the feral population [27,28,29]. Legge et al. (2020) was the first to summarise the effects of pet cat predation in Australia but did not focus on roaming behaviours and movements [11]. Owned cats roaming near natural areas have demonstrated higher predation levels than those in more urban areas [30]. More area-specific studies of free-roaming cat behaviour are needed in areas that encroach or are close to natural wildlife habitats and conservation areas, such as National Parks where the consequences of predation on wildlife are presumably more severe.

The aim of this study was to quantify cat roaming behaviour and assess potential for wildlife predation as indicated by spatial and temporal overlaps between wildlife and cats in two Sydney local government areas (LGAs), Campbelltown City Council (CTCC) and the Blue Mountains City Council (BMCC), to fill existing knowledge gaps and provide area-specific data. BMCC contains high levels of National Park land compared to CTCC, which has more than double the population of BMCC [31,32,33,34]. Based on current known demographics of the two LGAs, it was hypothesised that the density of free-roaming cats would be higher in CTCC than BMCC. Conversely, the observed wildlife numbers were hypothesised to be higher in the BMCC than CTCC.

## 2. Materials and Methods

### 2.1. Study Sites

The New South Wales (NSW) RSPCA initiated a new project ‘Keeping Cats Safe at Home (KCSAH)’ in 2021 which will run until 2025 [35]. Eleven councils in NSW, Australia are working with the project including CTCC and the BMCC [35]. Through a multidisciplinary approach, the project aims to encourage and support cat owners in the participating council areas to prevent their cats from roaming to reduce wildlife impacts and keep cats safe [35,36]. The data from this study will be utilised as a baseline prior to the implementation of interventions as a part of the RSPCA NSW KCSAH project.

The Blue Mountains (BM) and Campbelltown (CT) are two diverse LGAs within Greater Sydney, NSW Australia. The BM contains approximately 78,121 people in 35,139 private dwellings with a density of around 55 people per square kilometre (78,121/1430 km^2^), and CT has approximately 176,519 residents in 63,062 dwellings with a much higher density (~10 times higher) of around 566 people per square kilometre (176,519/312 km^2^) [31,32,33,34]. Recent comprehensive data on cat numbers and ownership is lacking in Australia, however, one study in 2009 found that there were high proportions of cat ownership in CT (≥30%) and another from 2018 recorded that 54% of survey participants in the BM had owned cats [37,38].

**Figure 1:**
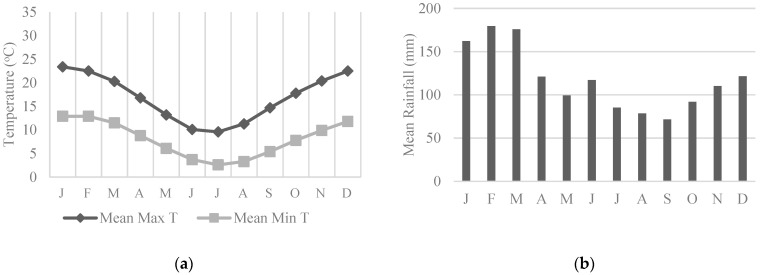
The Blue Mountains weather trends adapted from the Bureau of Meteorology (BOM). Data collected at Farnells Rd, Katoomba weather station: (**a**) Mean monthly temperatures (minimum and maximum), data collected 1907–2022 [39,41]; (**b**) Mean monthly rainfall, data collected 1885–2022 [40].

**Figure 2:**
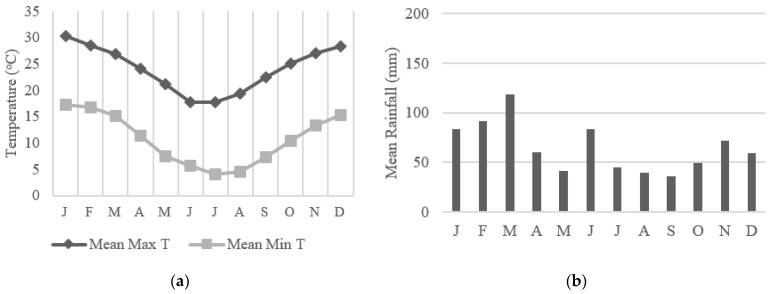
Campbelltown weather trends adapted from the BOM. Data collected at Mount Annan, Campbelltown weather station: (**a**) Mean monthly temperatures (minimum and maximum), data collected 2006–2022 [42,43]; (**b**) Mean monthly rainfall, data collected 2006–2022 [44].

National Park covers 74% of the BM (1058.2/1430 km^2^), and whilst a more built-up suburban area, CT also contains the Dharawal National Park which covers 20.9% of the LGA (65.08/312 km^2^) [31,32]. General weather conditions in the BM are more temperate than CT (up to 7 °C lower) with rainfall ranging between 70 and 180 mm per month (Figure 1a,b) [39,40,41]. CT is comparatively warmer and drier on average than the BM throughout the year (Figure 2a,b) [42,43,44].

### 2.2. RSPCA NSW Survey

The RSPCA NSW conducted an online survey in 2021 titled ‘What do you think about cats?’ available to anyone residing in NSW, Australia as part of their KCSAH project [45] (in press). It was advertised on their website, on their social media channels (Facebook and Instagram), and shared by veterinary practices, other rehoming organisations, and local councils. A total of 8708 people completed the survey, of which 683 were in the BM and 515 in CT. Within the survey, household participants were asked if they would like to volunteer to host a wildlife camera on their property. Once the BM and CT were selected as LGAs for monitoring, those households that responded ‘yes’ were contacted via email to provide consent and physical addresses. One hundred volunteer locations (50 BM and 50 CT) were randomly selected using Microsoft 365 Bing 3D maps in Microsoft Excel (Microsoft, Redmond, WA, USA).

### 2.3. Roaming Behaviours

#### 2.3.1. Motion-Capture Cameras (Indirect Observations)

**Figure 3:**
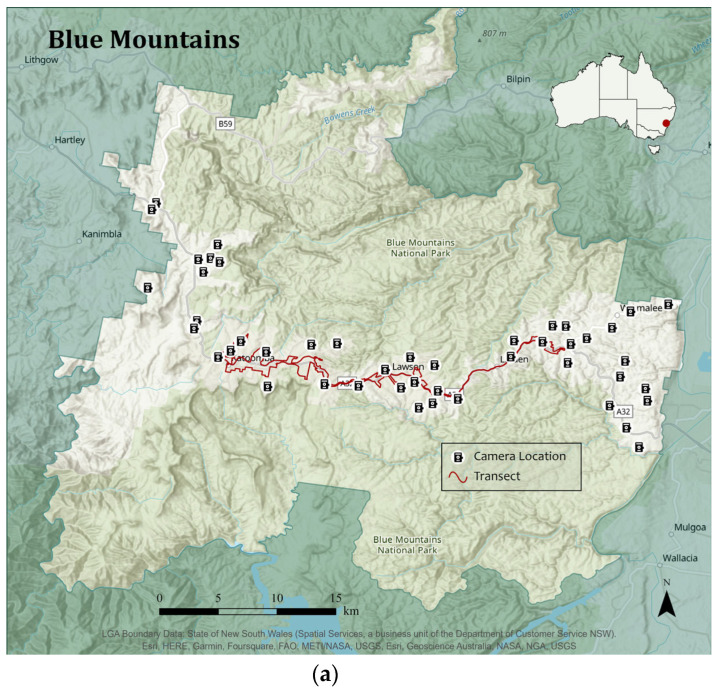

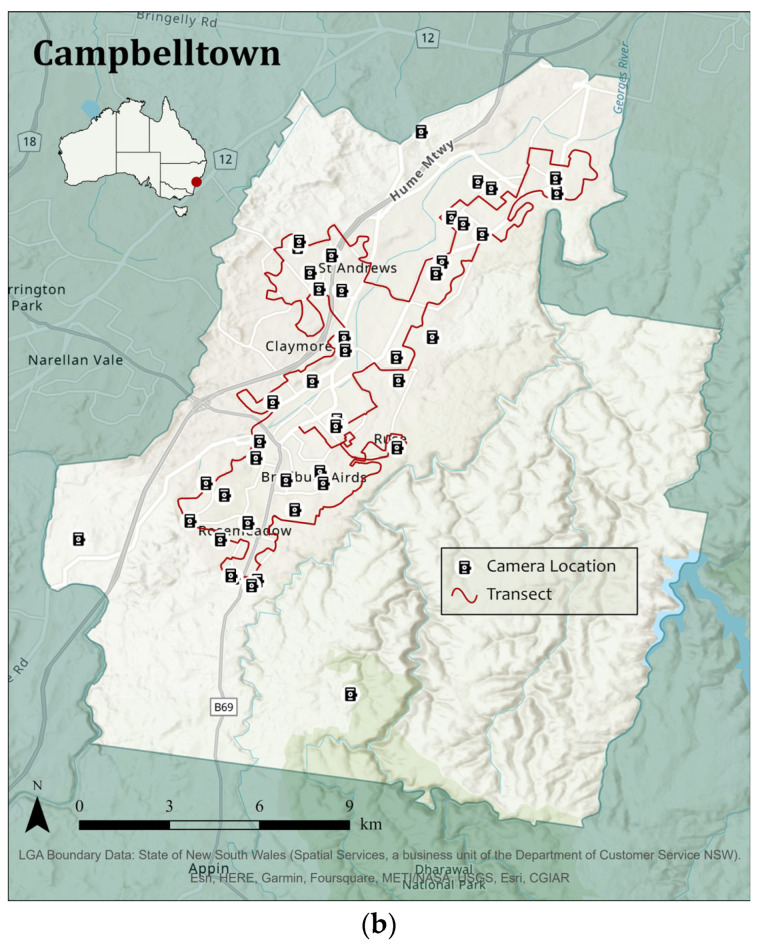
Camera locations and transect drive routes in: (**a**) The Blue Mountains (BM); (**b**) Campbelltown (CT). Maps were created using ESRI 2022. ArcGIS Pro: Release 2.9. Redlands, CA: Environmental Systems Research Institute.

A total of 100 heat-in-motion capture cameras (Swift 3C wide-angle, Outdoor Cameras Australia, Toowoomba, QLD, Australia) were used; 50 in the BM LGA and 50 in the CT LGA (Figure 3). Cameras were placed on private property of volunteers and remained there for approximately two consecutive months (February–April 2022).

As seen in Table 1, the weather during the study period in the BM was relatively wet, especially during March with a total of 685.8 mm recorded, as was CT which also had the highest rainfall of the study period during March and a total of 452 mm recorded [40,44]. Compared to the monthly means in Figure 1a and Figure 2a, maximum temperatures were slightly lower than average for these months in both LGAs, and the minimums were relatively similar [39,41,42,43].

**Figure 4:**
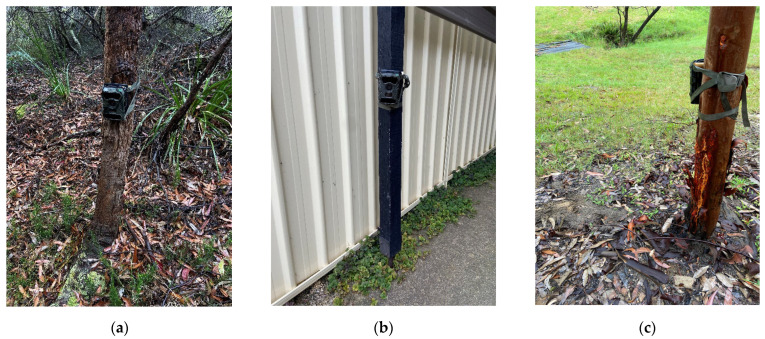
Cameras set up in the field on volunteer properties at approximately 90 cm above ground level: (**a**) Camera on a tree in the BM LGA on private property encroaching on wildlife habitat; (**b**) Camera on a car port in the CT LGA; (**c**) Camera on a tree looking out at potential cat trails in the BM. BM = Blue Mountains, CT = Campbelltown, LGA = local government area.

Cameras were attached vertically to an object already available on the property (for example a tree or fence post; Figure 4). The cameras were attached at approximately 90 cm above ground level and angled downwards. When outside, cats most often follow pre-formed tracks and fence lines when roaming. Therefore, locations on the volunteer properties that contained pre-formed tracks were ideally chosen to increase the likelihood of capturing free-roaming cats [46]. Cameras placed on a track were set up with an angle of incidence of approximately 22° to the track, whilst those pointed at a direct source (e.g., water source, and hole in the fence) were aimed directly at the source. The camera settings were set to: Passive Infrared (PIR) sensitivity—High; photo captures per trigger—3; interval between triggers—0 s; and image size—8 megapixels.

#### 2.3.2. Transect Drives (Direct Observations)

Transect drives are a quick and low-cost method that involves following a recorded path and documenting target objects to measure the distribution across a certain area [7,47]. After the collection of the 100 cameras, transects were completed in both the BM and CT LGAs. The transect drive for each LGA was determined on route during the first day and recorded using the MotionX—GPS app (Santa Cruz, CA, USA) on a mobile phone. Transects were approximately 80 km in length (excluding any highway travel wherein a 30 km/h speed limit was not safe and where there were no residential households). Following this route, the same transects were completed once a day at approximately the same time on four separate days over a seven-day period in each LGA with weather conditions generally clear, except for rainfall on 20 and 22 April in the BM (Table 2).

Transects were conducted from 2:30 pm to 5:30 pm AEST four times in each LGA (8 transects in total). Three to four people conducted the transect drives, with one driver and either two or three spotters to make observations. A physical tally sheet was used to manually record the data collected by the spotters including date, start and finish times, and the presence of free-roaming cats. Cats that were unable to roam (e.g., those observed inside a building through a window/door, those outside on a leash/lead, those outside but confined in an enclosure) were not recorded. Approximate distance from the vehicle of each identified cat was also recorded in metres. The vehicle’s headlights were switched on to assist with visibility, particularly after 5 pm when the levels of natural light decreased.

### 2.4. Data and Statistical Analysis

All images taken by the cameras were downloaded and individually viewed and tagged using the ExifPro 2.1 image-tagging program (Bad Kreuznach, Rheinland-Pfalz, Germany). The following tags were used if objects were identifiable: ‘BAN’ (bandicoot), ‘BIRD’, ‘CAT’, ‘DDOG’ (domestic dog *C. familiaris*), ‘DOG’ (other non-domestic dog, i.e., wild dog or dingo), ‘FOX’ (*Vulpes vulpes*), ‘GOANNA’, ‘GUPIG’ (guinea pig), ‘HOR’ (horse), ‘HUMN’ (human), ‘LIZ’ (lizard), ‘MAC’ (macropods including kangaroos and wallabies), ‘POS’ (possum), ‘RAB’ (rabbit/hare), ‘SMLMAM’ (any other mammal smaller than a bandicoot, including invasive rats and mice, bush rats and dunnarts) and ‘VEH’ (vehicles including cars, postmen, and trucks). If unidentifiable, images were tagged using the following: ‘NIL’ (nothing in the image), ‘UNK’ (too dark to determine if a target object was present), or ‘BADCAM’ (problem with the camera deeming the image compromised). The tagged images were then loaded into the R Studio program (Boston, MA, USA) in their groups of 3 images per trigger. An event was defined in R Studio as every group that was captured 60 s or greater apart from the last; this would count as one observation. These events were counted, and the unidentifiable images were removed along with any missing data. The clean dataset was compiled into a csv file by location, camera number, and tag.

A Shapiro–Wilk test was conducted to compare the cat events observed and test for normality. Even if data was proven not to be normally distributed, a Welch Two Sample *t*-test was run for a small sample size. The Shapiro–Wilk test and the Welch Two Sample *t*-test were also run to compare wildlife events (including the tags: ‘BAN’, ‘BIRD’, ‘DOG’, ‘FOX’, ‘GOANNA’, ‘LIZ’, ‘MAC’, ‘POS’, and ‘SMLMAM’).

The overlap package in R Studio was used to determine the coefficient of overlapping of ‘CAT’ and ‘POS’, ‘BAN’ and ‘SMLMAM’. Dhat4 estimator was used due to the high sample sizes.

The transect drive data was entered manually into a Microsoft Excel file and loaded into R Studio for analysis. Three separate models were tested: (i) half-normal with cosine adjustments, (ii) uniform with cosine adjustments, and (iii) hazard-rate with simple polynomial adjustments. Akaike information criterion (AIC) was used to determine which would be the most suitable mode, with the lowest AIC number indicated the best-fitting model. The distance package in R Studio was used to estimate cat encounter rates (ER = number of cats/km), density (density of cats/ha) and abundance (estimated cat population), with associated levels of precision included in the form of standard error (SE). As the transects were conducted through residential areas only, cat abundance was extrapolated based only on the residential areas of the LGAs (1604 ha in the BM and 10,000 ha in CT) that were provided by the councils. Coefficient of variation (CV) and 95% confidence intervals (LCL and UCL) were also included. An unpaired *t*-test was conducted to compare the cats observed for each day between LGAs.

For all analyses, a *p* value < 0.05 was considered significant.

## 3. Results

### 3.1. Motion-Capture Cameras (Indirect Observations)

Of the 100 cameras, 87 (44 BM and 43 CT) successfully captured 30,029 events (14,277 BM and 15,752 CT) (Table A1 in Appendix A). Thirteen cameras (six BM and seven CT) were not included for various reasons including equipment error, human error, or public disturbance/removal. Of the 30,029 events, 57.9% (*n* = 17,380) were animals tagged under 14 categories (Table 3). The remaining 42.1% (*n* = 12,649) contained other moving objects (‘HUMN’ and ‘VEH’).

Cats were captured in 1289 events in the BM and 4118 in CT on 83.9% of the cameras (BM = 34/44 cameras and CT 39/43). A total of 195 individual cats were recorded (BM = 79 and CT = 116 cats). Using the occupancy modelling in R Studio, these results lead to predict that cats would appear at 76% of sites in the BM, and they would be detected on camera around 25% of the time when present (Table 4). In CT, cats were predicted to appear at 83% of sites and be detected 33% of the time when present (Table 4).

**Figure 5:**
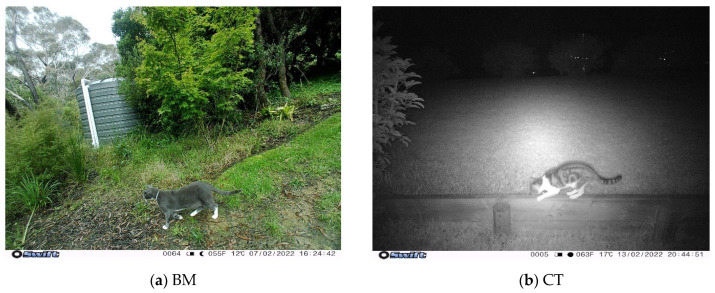
Roaming cats (*F. catus)* on the motion-capture cameras: (**a**) Cat with a collar during the day on a BM camera; (**b**) Cat at night on a CT camera. BM = Blue Mountains, CT = Campbelltown.

**Figure 6:**
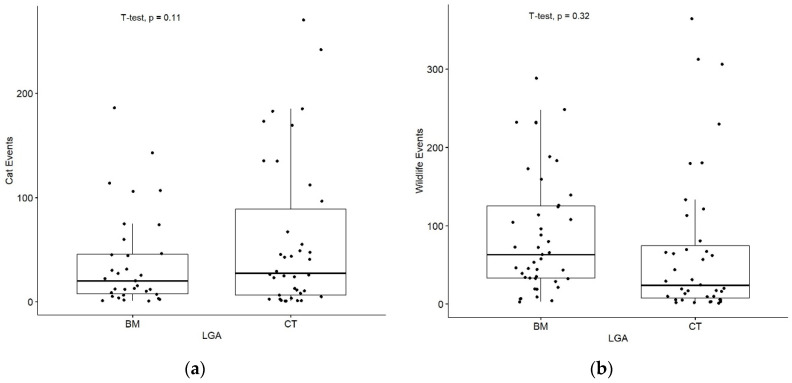
Indirect (camera) events: (**a**) Cat (*F. catus*) events with 1 true outlier removed in CT for better visualisation—median cat events observed 20.0 (BM) and 27.5 (CT). There was no significant difference between these local government areas (*p* = 0.11); (**b**) Wildlife events with 1 true outlier removed in the BM—median events observed 63.0 (BM) and 24.0 (CT). No significant difference between the local government areas was found (*p* = 0.32). BM = Blue Mountains, CT = Campbelltown.

Cat roaming events captured per camera (for example in Figure 5) were higher in CT (mean events per camera = 60.76, median events per camera = 27.50, and total events = 4118) than the BM (mean = 37.91, median = 20.00, and total events = 1289), but this difference was not statistically significant between the two LGAs (*p* = 0.11, Welch Two Sample *t*-test; Figure 6a). Conversely, wildlife events captured per camera were higher in the BM (mean = 88.33, median = 63.00, and total events = 5580) compared to CT (mean = 69.15, median = 24.00, and total events = 2697); however, this difference was also not significant (*p* = 0.32, Welch Two Sample *t*-test; Figure 6b).

**Figure 7:**
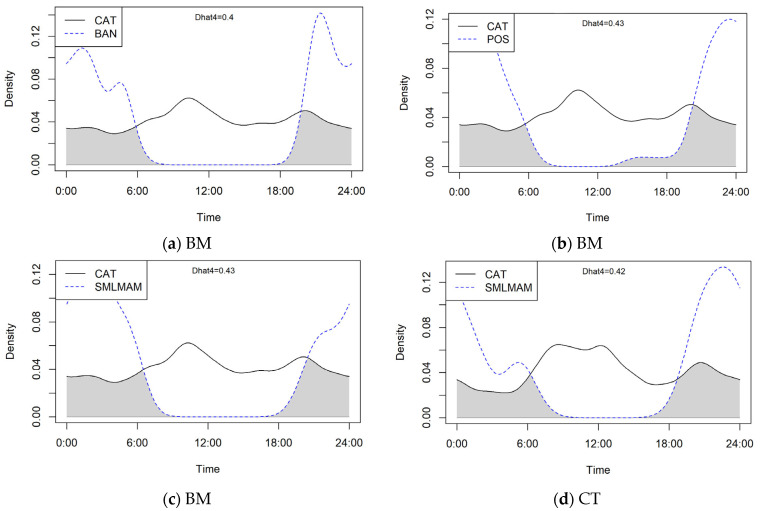
Activity overlaps graphed from motion-capture camera data: (**a**) Cats and bandicoots (BAN) in the BM; (**b**) Cats and possums (POS) in the BM; (**c**) Cats and small mammals (SMLMAM) in the BM; (**d**) Cats and small mammals (SMLMAM) in CT. BM = Blue Mountains, CT = Campbelltown.

**Figure 8:**
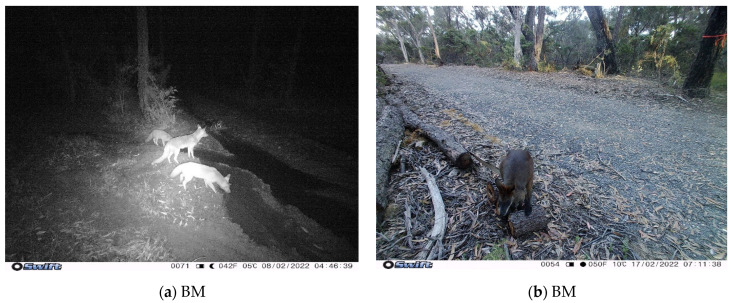
Wildlife captured on the motion-capture cameras in the Blue Mountains (BM): (**a**) Foxes (*Vulpes vulpes*) at night; (**b**) Swamp wallaby (*Wallabia bicolor*) in daylight.

Cats were indirectly observed (i.e., captured on camera) throughout all hours of the day, however, cats were most often observed at around 9:30 am and 8:00 pm in the BM, and 7:00 am and 12:00 pm in CT (Figure 7). The temporal movements of cats were also plotted against wildlife observations to calculate overlap (Figure 7). For BM, 1252 cat observations were plotted against (a) 228 bandicoot, (b) 416 possum, and (c) 161 small mammal observations. For CT, 4099 cat and (d) 6 small mammal records were graphed (note: no bandicoots or possums were observed in CT). All four graphs show a general trend of overlapping activity between 6:00 pm and 7:00 am. Possums in the BM displayed more activity with overlap from around 1:00 pm. Small mammals in both the BM and CT also seemed to show more activity in the morning until around 9:00 am. The coefficient of overlapping was similar for all four graphs measuring between 0.4 and 0.43, suggesting an estimated overlap of 40% (a), 43% (b), 43% (c), and 42% (d) for cat and wildlife activity. Additionally, cats can be seen to be active throughout the entire day at both sites. Larger mammals (for example in Figure 8) were also observed.

### 3.2. Transect Drives (Direct Observations)

**Figure 9:**
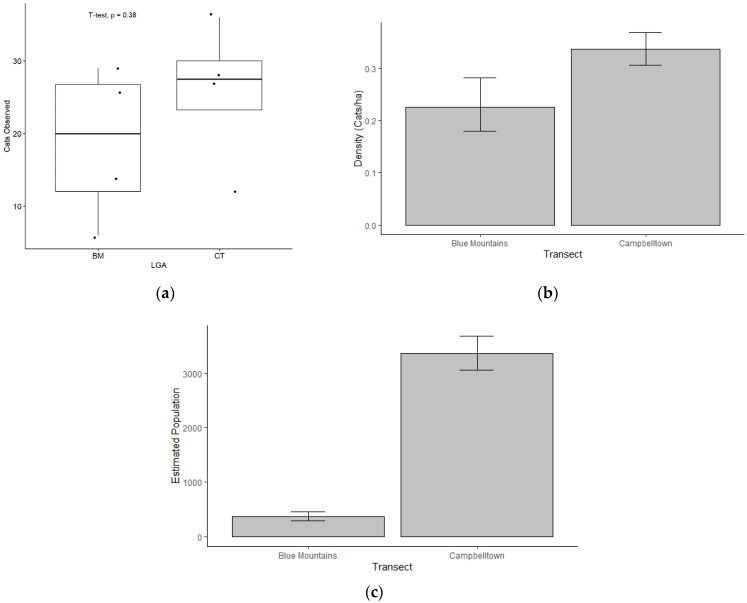
Direct (transect drive) cat observations for the two LGAs, the Blue Mountains (BM) and Campbelltown (CT): (**a**) Number of cats observed across four study days in each LGA; mean cats observed in the BM (18.8) and CT (25.8) was not significantly different (*p* = 0.38); (**b**) Densities of cats per hectare (cats/ha); (**c**) Overall estimated abundance of free-roaming cats (total number of cats).

During the direct observations made on eight separate transect drives (four in each LGA), a total of 178 free-roaming cats were observed. Of these, 75 were in the BM and 103 in CT (Table A2 in Appendix A). In the BM, each observation during the transect drives recorded one cat at a time. Two cats were recorded together on one occasion in CT. Overall, the number of cats observed was higher in CT than the BM but was not statistically significant (*p* = 0.38; two sample *t*-test; Figure 9a). The ER (encounter rate of cats per km) was higher in CT (1.40) compared to the BM (0.94) (Table A3 in Appendix A). Overall, the standard error (SE) of the mean was low (BM 0.00, and CT 0.03), indicating little to no random error between sampling events, suggesting high precision in the monitoring model (Table A4 in Appendix A). The density of cats was estimated to be 0.31 cats/ha in CT compared to 0.21 cats/ha in the BM (Figure 9b), producing an overall estimated abundance of 361 free-roaming cats in the residential areas (1604 ha) of the BM and 3365 free-roaming cats in the residential areas (10,000 ha) of CT (Figure 9c).

Moreover, the SE for density was close to 0 (0.02 for both the BM and CT), implying no random error and a high precision model. The narrow limits between the upper and lower 95% confidence intervals (BM 0.15–0.29, and CT 0.24–0.39) further indicate the precision of the density data (Table A4 in Appendix A). However, SE for abundance was expectedly high (24.65 for the BM and 157.21 for CT; Table A5).

## 4. Discussion

The data from this observational study is the first to quantify free-roaming cats in the two LGAs of Greater Sydney, the Blue Mountains and Campbelltown. Both direct (transect drives) and indirect (motion-capture cameras) observations will provide the basis for the RSPCA NSW ‘Keeping Cats Safe at Home’ program in these LGAs and help to guide future management tools for free-roaming cats. This data may also enhance further research in the field of cat monitoring.

The use of motion-capture cameras is valuable for monitoring cat roaming as well as cat-wildlife interactions [7,54,55]. Only 83% of cameras successfully captured images (44 BM and 43 CT), compromising the reliability of results, but still enabling many events to be captured (30,029) including free-roaming cats (5407). The high number of cat observations collected in Campbelltown (4118) and the Blue Mountains (1289) enabled us to make a good foundational quantification to estimate cat numbers in each LGA, as well as to make other comparisons. Although accuracy might be questioned due to the definition of an ‘event’ meaning if captured after 60 s, the same cat would be identified as a separate event, the minimum number of individual cats was also recorded (BM = 79 and CT = 116 cats). The cat events (cameras) and density (transects) were higher in Campbelltown than the Blue Mountains as hypothesised, yet were not significant, despite the large difference in human population density (approximately 10 times higher in CT). Elizondo and Loss (2016) found no relationship between cat abundance and human population density nor between cat abundance and urban development intensity [54]. However, Finkler, Hatna and Terkel (2011) found that adult and kitten densities somewhat depended on socio-demographics, whilst Flockhart, Norris and Coe (2016) reported highest cat abundance in high-density low-income residential neighbourhoods [47,55]. Ownership status could not be determined for most cats in the current study, unless they were wearing a collar, meaning like other observation studies they could not be categorised further than domestic cat (*F. catus*) [56].

It is important to note that the current study was predominately carried out during the period of intensified cat oestrus in temperate regions of the southern hemisphere (approximately September to March) [57]. This means that our overall results may have been skewed to indicate higher levels of cat free-roaming due to migration associated with mating and increased libido in cats during this period. Therefore, results may differ from other times of the year when migrations may be lower, and further research would be required to evaluate the effect of seasonality.

The number of wildlife events captured was higher in the Blue Mountains (5580) than Campbelltown (2697) but was not statistically significant despite BM containing more natural habitat. Future research is needed to explain this trend, and although speculative at this stage, reduced numbers of wildlife in CT due to wildlife predation by cats is possible. Wildlife predation has been identified as a serious threat posed by free-roaming cats [4]. Further studies could focus on these relationships in greater detail to continue to assess cat behaviour and determine if levels of their wildlife predation are truly detrimental to the environments in which they roam, and whether ecosystem balances and biodiversity are directly impacted, particularly in these areas of Greater Sydney now that baseline data has been collected. Wildlife events in CT may also have included more generalist and urban adaptor species, flying bird species, ‘SMLMAM’ small mammals included under wildlife, and other tags less affected by the presence of cats, such as ‘DOG’ and ‘FOX’. There were also outlier cameras that may have contributed to this lack of statistical significance with high numbers recorded of one or more certain species; for example, camera 21 (CT) captured 234 guinea pig events, and camera 74 (BM) captured 1025 rabbit events.

The findings of this study have potentially important implications for cat management interventions with a One Health focus. One Health is an all-encompassing term that considers animal, human, and environmental health. The health of each aspect affects the others, particularly in terms of economic impacts and disease burden [58]. In addition to predating wildlife and affecting wildlife behaviour, free-roaming cats can carry pathogens affecting both other cats and non-feline animals including livestock [59]. For example, cats are the only definitive host of *Toxoplasma gondii*—the causative parasite of toxoplasmosis—and high cat densities are correlated with declining wildlife populations because of this disease [13,18]. *T. gondii* is prevalent in Australian wildlife, causing disease in many native mammal and bird species, and contributing to high mortality [17,19]. Cats can affect humans by spreading zoonotic diseases (e.g., cat scratch fever caused by the bacterium *Bartonella henselae*), injuring them, and displaying nuisance behaviour including spraying and fighting [30,60,61,62,63]. Another zoonotic disease that may be spread by free-roaming cats is visceral larva migrans, caused by a gastrointestinal ascarid (*Toxocara cati*), usually in young children following ingestion of contaminated soil [64]. The ability to accurately quantify cat populations is essential when monitoring and evaluating interventions to reduce the impacts of free-roaming cats on One Health. A further issue is the effect of cat control on mesopredators. For example, invasive rats have been known to increase with the removal of larger predators, such as cats [65,66,67]. However, smaller mammals, and in particularly fast-moving mammals, are difficult to identify on camera images, particularly when they are positioned to capture a different target (i.e., larger mammals, such as cats) [7].

The motion-capture cameras required little physical labour apart from setting up and had the benefit of continuous observation. However, the high number of cameras (100) made them relatively expensive (approximately $24,000 AUD total) including batteries, Secure Digital (SD) cards, chargers, shipping, and storage. It is general knowledge that cats are often crepuscular and nocturnal hunters, however, our results were not consistent with this as cats were observed throughout all hours of the day with peaks at around 9:30 am and 8:00 pm in the BM, and 7:00 am and 12:00 pm in CT [13]. This could be due to a high population of owned cats in these areas demonstrating free-roaming behaviours that might vary from feral and unowned cats. This has also been reported in other urban areas, where approximately 96.5% of individual cats were captured on cameras during both day and night [54]. The night range (10 m) was less than during the day so potential night roaming cat events might have been missed, again affecting the accuracy of the results. Further, although manual tagging of the images ensured high accuracy of identifying the events and the objects causing them, this aspect was labour intensive. Future studies using image tagging should seek to solve this issue by exploring other avenues, such as deep learning and artificial intelligence (AI). Other researchers have demonstrated that with the same 96.6% accuracy as humans, deep learning can automatically identify 99.3% of large image datasets [68].

The transect drives were a relatively quick (3 h per transect) method to directly observe free-roaming cat numbers with little to no cost equipment-wise, particularly in comparison to the cameras. They covered a large distance of residential area (80 km) in each LGA. The transects produced sample data for cat density which was estimated to have high precision amongst sampling events. They were, however, high-cost in labour with one driver and two to three people observing manually per drive. Furthermore, the timing of the transects (2:30 pm–5:30 pm) were selected to fit within the working hours of the council staff and volunteers. This timing does not align with when cats are more likely to be roaming [13]. Therefore, estimates of abundance may be underestimated. However, as this data is baseline, further transects conducted at the same times will be able to determine any changes in roaming behaviours.

## 5. Conclusions

This study provides a baseline for the RSPCA NSW Keeping Cats Safe at Home project for two LGAs in Greater Sydney, Campbelltown and the Blue Mountains, to be used for further studies. Observations found no statistical difference between cat events and wildlife events in the two LGAs. However, both cat numbers and density were higher in Campbelltown, and wildlife numbers were higher in the Blue Mountains. Cats were observed throughout both day and night, however, they were mostly observed roaming during early afternoon. Remote sensing cameras seem to be the most cost-effective tool to monitor free-roaming domestic cats, particularly in the context of wildlife conservation, and One Health interventions.

## Figures and Tables

**Table 1:** Weather conditions during the camera study period (February–April 2022) comparing the two local government areas (LGAs), Blue Mountains and Campbelltown. Taken from the Bureau of Meteorology [39,40,41,42,43,44].

LGA	Weather Condition	February	March	April
Blue Mountains	Total Rainfall (mm) ^1^	246.9	685.8	215.6
	Mean Max T (°C) ^2^	20.6	18.9	17.3
	Mean Min T (°C)	12.6	12.3	9.6
Campbelltown	Total Rainfall (mm)	161.2	452	158
	Mean Max T (°C)	27.2	25.6	23.6
	Mean Min T (°C)	16.3	16.2	12.8

^1^ Rainfall is in millimetres (mm). ^2^ T = temperature in degrees Celsius (°C).

**Table 2:** Weather conditions for the transect study days by local government area (LGA) site collated from the Bureau of Meteorology Katoomba (the Blue Mountains) and Mount Annan (Campbelltown) weather stations [48,49,50,51,52,53].

LGA	Date	Rainfall (mm) ^1^	Max T (°C) ^2^	Min T (°C) ^2^
Blue Mountains	19 April 2022	0.0	21.8	13.4
	20 April 2022	12.4	18.2	7.8
	21 April 2022	0.0	17.4	6.0
	22 April 2022	2.0	11.8	8.1
Blue Mountains Average		3.6	17.3	8.8
Campbelltown	21 April 2022	0	23.7	8.3
	22 April 2022	0	20.1	12.0
	26 April 2022	0	20.2	13.2
	27 April 2022	0	18.7	13.0
Campbelltown Average		0	20.7	11.6

^1^ Rainfall is in millimetres (mm). ^2^ T = temperature in degrees Celsius (°C).

**Table 3:** Summary of identifiable animal camera tags for two local government areas (LGAs), Blue Mountains (BM) and Campbelltown (CT).

LGA	BAN	BIRD	CAT	DDOG	DOG	FOX	GOANNA	GUPIG	HOR	LIZ	MAC	POS	RAB	SMLMAM	Total
BM	230	4285	1289	1303	7	216	3	0	0	1	245	417	1025	176	9197
CT	0	2566	4118	1111	0	78	0	235	4	3	43	1	18	6	8183
Total	230	6851	5407	2414	7	294	0	235	4	4	288	418	1043	182	17,380

‘BAN’ (bandicoot), ‘BIRD’, ‘CAT’, ‘DDOG’ (domestic dog), ‘DOG’ (other dog, e.g., wild dog/dingo), ‘FOX’, ‘GOANNA’, ‘GUPIG’ (guinea pig), ‘HOR’ (horse), ‘LIZ’ (lizard), ‘MAC’ (macropods), ‘POS’ (possum), ‘RAB’ (rabbit/hare), and ‘SMLMAM’ (any mammal smaller than a bandicoot).

**Table 4:** Occupancy (predicted chance of cats occurring in the area) and detection (predicted chance of observing a cat in the area) of free-roaming cats in the Blue Mountains and Campbelltown LGAs. LGA = local government area, SE = standard error, Q1 = lower quartile, Q3 = upper quartile.

Blue Mountains				
	Predicted	SE	Q1	Q3
Occupancy	0.7565806	0.06397333	0.6113989	0.859947
Detection	0.2544477	0.009194475	0.2368506	0.2728848
**Campbelltown**				
	**Predicted**	**SE**	**Q1**	**Q3**
Occupancy	0.8260867	0.05588564	0.6890605	0.9105652
Detection	0.3303644	0.009463841	0.3120863	0.3491696

## Data Availability

No new data other than those presented were created or analyzed in this study. Data sharing is not applicable to this article.

## Appendix A

**Table A1:** Collation of all successful cameras and events tagged. Cameras are separated by local government area (Campbelltown and the Blue Mountains) and events for each camera (numbered) are identified by tag: ‘BAN’ (bandicoot), ‘BIRD’, ‘CAT’, ‘DDOG’ (domestic dog), ‘DOG’ (other dog, e.g., wild dog/dingo), ‘FOX’, ‘GOANNA’, ‘GUPIG’ (guinea pig), ‘HOR’ (horse), ‘HUMN’ (human) ‘LIZ’ (lizard), ‘MAC’ (macropods), ‘POS’ (possum), ‘RAB’ (rabbit/hare), ‘SMLMAM’ (any mammal smaller than a bandicoot), and ‘VEH’ (vehicles).

Campbelltown (CT)																	
Cameras	BAN	BIRD	CAT	DDOG	DOG	FOX	GOANNA	GUPIG	HOR	HUMN	LIZ	MAC	POS	RAB	SMLMAM	VEH	Total
**1**		69	112							249							430
**2**		3	44	95						433							575
**3**		65		11		2				32				1			111
**4**		64	47	1						61							173
**5**		19	183	5						175							382
**6**		364	1					1		217							583
**7**		56	26	3		1				386							472
**10**		62	13	5						25							105
**11**			55							35							90
**12**		230	6	2						267							505
**13**		29								9							38
**14**		3	43	125						156							327
**15**		9		54						48							111
**17**		31	5	1						53							90
**18**		2	29							35							66
**19**			3							40							43
**20**		5	8	126						236							375
**21**		16	173					234		163							586
**22**		2								33							35
**23**		17	169							53							239
**24**		179	1							151		1					332
**25**		65	185	3		1				42							296
**26**		309	242	1		3				47							602
**27**			1809							9							1818
**28**		113	24	371						706							1214
**29**		3	23	2						145						66	239
**30**		17	49	1						309							376
**31**		10	1	4						51							66
**32**		10	45							171							226
**33**		3	11							303	3						320
**34**		1	2	3						97							103
**35**		44	1							101							146
**36**		79	135			1				58							273
**38**		20	135	1						48							204
**39**		121	41	4						191							357
**40**			1							18							19
**41**		5	96							123							224
**43**		2	270	95		3				845							1215
**44**		306	26							540							872
**46**		133	10							78							221
**48**		24	67	158					4	723				6			982
**49**		13	25	1						40							79
**50**		63	2	39		67				1		42	1	11	6		232
**CT** **Total**	**0**	**2566**	**4118**	**1111**	**0**	**78**	**0**	**235**	**4**	**7503**	**3**	**43**	**1**	**18**	**6**	**66**	**15,752**
**Blue Mountains (BM)**																	
**54**		7			1					8		11					27
**55**		25	12			6				57		37	16		4		157
**57**		122	10	2						69			2		2		207
**58**		4	12	5						204			2				227
**59**		1	2							35		2	1				41
**61**		159								373							532
**62**		23	15	48						166			13		17		282
**64**		12	45	1		5				31		42	6				142
**65**		101	13	1						106			59		28		308
**66**		35	12	104						168			1		37		357
**67**		124	30							204							358
**68**		65	27	1		26				150		2	5		6		282
**69**		24	46			1				157			14		4		246
**70**	4	1740	74	12		6				798			2		30		2666
**71**		8		7		70				15		2					102
**72**		93	25	121		2				55			13				309
**73**		11	106	110	1	22				328							578
**74**		168	60			77				5				1025	3		1338
**75**		58		683						747							1488
**76**		229	20	189						78			3				519
**77**		29	44	2						164							239
**78**		72	22							42							136
**79**		165	7							168			2		16		358
**80**		4			1							2					7
**81**	5	111	6	2	3					147			20				294
**82**		231		5						59							295
**83**		32		1						7							40
**84**		6	2										3				11
**85**						1				1		43					45
**86**		8	3	1						8			25				45
**87**	1	16								9		5	12		1		44
**88**	11	10	31									62	13				127
**89**		14	5	1	1					2	1		22		1		47
**90**		41								9		22					72
**92**		219	186							65			12		1		483
**93**		1	9							93			2				105
**94**		151	75	7						105			22				360
**95**	9	36	143							71			67		2		328
**96**		46	114							162							322
**97**	178	41	20							115			67		2		423
**98**	11	5	1									15	1		1		34
**99**	2	17	107							94							220
**100**	9	3	1		1		3			5		11	12		21		51
**BM Total**	**230**	**4285**	**1289**	**1303**	**7**	**216**	**3**	**0**	**0**	**5080**	**1**	**245**	**417**	**1025**	**176**	**0**	**14,277**
**Total**	**230**	**6851**	**5407**	**2414**	**7**	**294**	**3**	**235**	**4**	**12,583**	**4**	**288**	**418**	**1043**	**182**	**66**	**30,029**

**Table A2:** Direct observational free-roaming cat data collected during the transect drives collated by distance and site (Blue Mountains or Campbelltown). LGA = local government area.

LGA	Distance (m) *	Free-Roaming Cats
Blue Mountains	0	3
	2	1
	3	1
	5	12
	7	2
	10	25
	15	11
	20	8
	25	6
	30	3
	35	1
	50	1
	60	1
Blue Mountains Total		75
Campbelltown	2	1
	3	1
	4	1
	5	6
	7	4
	8	6
	10	30
	12	2
	15	28
	18	3
	20	14
	25	3
	30	4
Campbelltown Total		103
Grand Total		178

* Distance in metres (m) observed from the vehicle.

**Table A3:** Summary data of cat transects completed in April 2022 in the Blue Mountains and Campbelltown local government areas (LGAs) adapted from R Studio output where: ‘Area’ sample area (ha) covered during the transect, ‘Length’ length of transect (km), ‘n’ number of cats identified, ‘ER’ encounter rate, ‘Mean’ *n* per observation, and ‘SE’ standard error.

LGA	Area (ha)	Length (km)	*n*	ER	Mean	SE
Blue Mountains	960	80	75	0.94	1.00	0.00
Campbelltown	960	80	112	1.40	1.09	0.03
Total	1920	160	187	1.17	1.05	0.02

**Table A4:** Estimate of cat density as calculated from cat transects completed during April 2022 within the two local government areas (LGAs) of the Blue Mountains (BM) and Campbelltown (CT). Adapted from R Studio output where: ‘Density’ density estimate (cats/ha), ‘SE’ standard error, ‘CV’ coefficient of variation, ‘LCL’ lower 95% confidence interval, ‘UCL’ upper 95% confidence interval, ‘DF’ degrees of freedom.

LGA	Density	SE	CV	LCL	UCL	DF
Blue Mountains	0.21	0.02	0.09	0.15	0.29	2.30
Campbelltown	0.31	0.02	0.08	0.24	0.39	3.14

**Table A5:** Estimate of cat abundance as calculated from cat transects completed during April 2022 within the two local government areas (LGAs) of the Blue Mountains (BM) and Campbelltown (CT). Adapted from R Studio output where: ‘Abundance’ total population estimate (extrapolated), ‘SE’ standard error, ‘CV’ coefficient of variation, ‘LCL’ lower 95% confidence interval, ‘UCL’ upper 95% confidence interval, ‘DF’ degrees of freedom.

LGA	Abundance	SE	CV	LCL	UCL	DF
Blue Mountains	361.41	24.65	0.07	288.67	452.49	2.82
Campbelltown	3364.77	157.21	0.05	3062.99	3696.27	45.55
